# Stress transfer patterns and local seismicity related to reservoir water-level variations. A case study in central Costa Rica

**DOI:** 10.1038/s41598-019-41890-y

**Published:** 2019-04-03

**Authors:** S. Ruiz-Barajas, M. A. Santoyo, M. B. Benito Oterino, G. E. Alvarado, A. Climent

**Affiliations:** 1 Dpto. Ingeniería Topográfica y Cartografía, Universidad Politécnica de Madrid, Madrid, Spain; 2 Instituto de Geofísica, Universidad Nacional Autónoma de México, Campus Morelia (México), Morelia, Mexico; 3 Área de Amenazas y Auscultación Sismológica y Volcánica, Instituto Costarricense de Electricidad (ICE), San José, Costa Rica

## Abstract

This study main aim was to analyse the spatio-temporal trend in seismicity recorded in the proximity of the Pirrís Reservoir (central Costa Rica), where impoundment for the purposes of filling the reservoir to its total volume (3,6 * 107 m^3^) started in 2011. We differentiated between the events that occurred before, during and after this filling operation. Using a seismic analysis, we sought to define and understand the effects which such reservoir operations have on seismic activity in the area. To this end, we evaluated the spatio-temporal evolution of Coulomb failure stress (ΔCFS) changes due to surface water load, and its correlation with seismicity. Overall, the results of this study provide a perspective of how the water load in the reservoir can affect the stress state in the close area. In our study case, we have detected: an increase in b-value after impoundment, an increment of rate for shallowest events (h ≤ 10 km), an increasing trend of higher magnitude events and a possible trigger effect on local faults. All these aspects could be useful to control the reservoir operations and to help in decision making in order to guarantee the safety of these critical emplacements.

## Introduction

For a number of decades, seismicity associated with human activities (specifically those linked to energy technologies) has been an issue of some considerable concern to the scientific community and society in general^1,2^. Cases of induced and triggered seismicity have risen dramatically in recent years, due to the increase in new subsurface exploration and energy exploitation projects (e.g., the case of Oklahoma)^3^. Among the most important anthropogenic activities which could trigger earthquakes are oil production, wastewater disposal, hydraulic fracturing, reservoir impoundment, geothermal operations, mining, and nuclear tests. Recently, a freely available database was published listing the different cases of induced and triggered earthquakes which have occurred around the world to date (HiQuake, Human-induced Earthquake Database). The database includes more than 700 anthropogenic projects proposed in scientific studies, such as earthquakes associated with human activities^4^ Although mining and water-reservoir impoundment are the anthropogenic activities that most commonly cause seismicity, in recent years the number of earthquakes associated with fluid-injection operations (i.e., fracking) has grown considerably^4,5^. Anthropogenic activities can alter the natural environment and perturb the stress state in different ways, depending on the type of activity and the tectonic-geological environment. Different authors have suggested ways of classifying the different types of induced and triggered seismicity, one example being the recent classification proposed by Doglioni^6^, based on the relationship between induced seismicity and hydrostatic and lithostatic pressures. This paper focuses on seismicity associated with impoundment operations. For the sake of simplicity, these effects will be referred to hereafter as reservoir-triggered seismicity (RTS), in line with the recommendation made by the International Commission on Large Dams (ICOLD) and a number of other authors^7^. The main aim of this study was to analyse the occurrence of this type of seismicity from a physically elastic perspective, focusing specifically on the Pirrís Reservoir in Costa Rica. The reason for this choice lay in the availability of a complete dataset, which included seismological data, physical characteristics of the dam, and data pertaining to the filling operations.

## Summary of previous studies addressing Reservoir-Triggered Seismicity

The first reported case of RTS dates from 1935 and concerned operations at the Hoover dam on Lake Mead in the USA. However, it was not until the early 1960s when interest in this phenomenon increased due to the rise in RTS cases. Among some of the most important cases across the globe, mention should be made of Xinfengjiang (China, 1962, M 6.2), Koyna-Warna (India, 1967, M 6.7), Oroville (California, 1975, M 5.7) and Kariba (Zambia-Zimbabwe border, 1963, M 6.2). Gupta^8^ presents a review of recent cases of RTS around the world, and a complete list of reported cases of RTS (169) by reservoir impoundment can be found in the HIQuake database (2018)^4^.

RTS is one of the most complicated cases of seismicity when it comes to understanding the driving physical mechanisms. This sort of seismicity is triggered by a complex interaction between diverse factors, such as reservoir size, field stress state, tectonic settings, hydrogeological conditions, time histories of the water level, etc. Various authors^9–13^ suggest that there are two main mechanisms responsible for RTS, linked to: (1) the added water weight in a reservoir; and (2) the water that seeps into cracks underground or along a fault. In the former case, the filling of a reservoir can increase the elastic shear stress and cause the rupture of nearby faults. In the latter case, the increase in and diffusion of pore pressure in the ground under and near the reservoir act to promote a decrease in effective normal stress. While the effect of water load is normally immediate, the pore pressure effect is delayed because water requires a time to flow through rock. This delay can cause some reservoirs to begin triggering earthquakes years after their initial filling. Furthermore, it is known that changes in a reservoir’s water level could affect the stability of nearby active faults, favouring the activation of normal faults in particular. A number of studies have investigated elastic (undrained) stress changes due to reservoir impoundment. For instance, Gough and Gough^14^ analysed the case of Lake Kariba (Zambia), and Beck^15^ examined stress changes at Lake Oroville (California). In a more recent work, Santoyo *et al*.^16^ presented an analysis of the effects of surface-water loads on the subsurface state of stress in the case of the Itoiz Reservoir (Spain). In all these studies, elastic stress changes were calculated without considering pore pressure changes due to the effects of Skempton’s pore-pressure parameter on the subsurface state of stress. In their theoretical analysis of stress and strength changes due to reservoir impoundment, considering both elastic stress and pore pressure changes due to diffusion, Bell and Nur^10^ found that the magnitude of strength change due to reservoir impoundment varied with the assumed permeability values and the location of permeability contrast. Subsequently, Simpson *et al*.^12^ considered both elastic (undrained) and diffusion effects to explain RTS, ascribing the initial seismicity near some shallow reservoirs, including the Monticello reservoir, to the elastic (undrained) effect alone^17^ This paper is structured as follows. Firstly, there is a description of the seismotectonic context and seismic data collected, followed by a complete analysis of seismicity. After performing a careful relocation process of the recorded seismicity, we then conducted an analysis of the spatio-temporal tend in events around the Pirrís Reservoir, considering three different periods, i.e., before, during and after the impoundment. Subsequently, the Coulomb static stress changes (ΔCFS) due to the impoundment operations were evaluated. For study purposes, spatial CFS and temporal pattern variations were both included. The paper concludes with an examination of the possible relationship between these ΔCFS and the seismicity recorded within the reservoir area.

### Seismotectonic setting

The Pirrís Reservoir is located in the Central Pacific zone of Costa Rica with a surface impoundment extension of 3.5 km in the E-W direction and 1.2 km in the N-S direction. This is one of the 13 largest hydroelectric power plants operated by the Costa Rican Electricity Institute (ICE, *Instituto Costarricense de Electricidad*). The Pirrís hydroelectric plant consists of a 113-m high dam, a 11-km long driving tunnel and a powerhouse operated by two Pelton turbines. The main characteristics of the reservoir are summarised in Table 1 below.

**Table 1:** Main characteristics of the Pirrís reservoir (*m a.s.l = meters above sea level).

Start of filling	2011
Reservoir Area	114 ha
Total Volume	3,6 * 10 ^7^ m^3^
Maximum level	1204 m a.s.l*
Minimum level	1160 m a.s.l*
Water column	80 m
Dam Height	113 m
Ridge length	270 m

**Figure 1:**
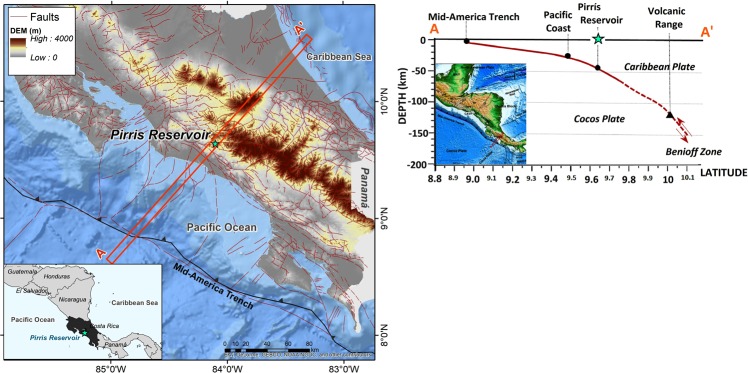
Location of the Pirrís reservoir with a national tectonic view of the Central America margin. This figure was generated by using the ESRI software, ArcGIS for desktop, version 10.3.1. (http://www.esri.com/).Source of the base map:Image of Esri, DeLorme, GEBCO, NOAA NGDC and other contributors. A profile of the subduction zone is included at the right side of the figure.

Due to the complexity of the reservoir’s tectonic environment, the seismicity in this area may essentially be associated with two main regimes, namely, active subduction and crustal local faults. Offshore Costa Rica, the Cocos Plate subducts beneath the Caribbean plate with subduction velocity rates that vary from 8.3 cm *yr*^−1^ in north-west to 9.3 cm *yr*^−1^ in the south-east of the country^18^. The Pirrís reservoir is located approximately 40 km above the interplate tectonic interface, where the Benioff zone earthquakes are produced by the interaction between the down-going oceanic plate and the continental plate. The Central region of Costa Rica is characterised by a low angle subduction regime (see Fig. 1).

**Figure 2:**
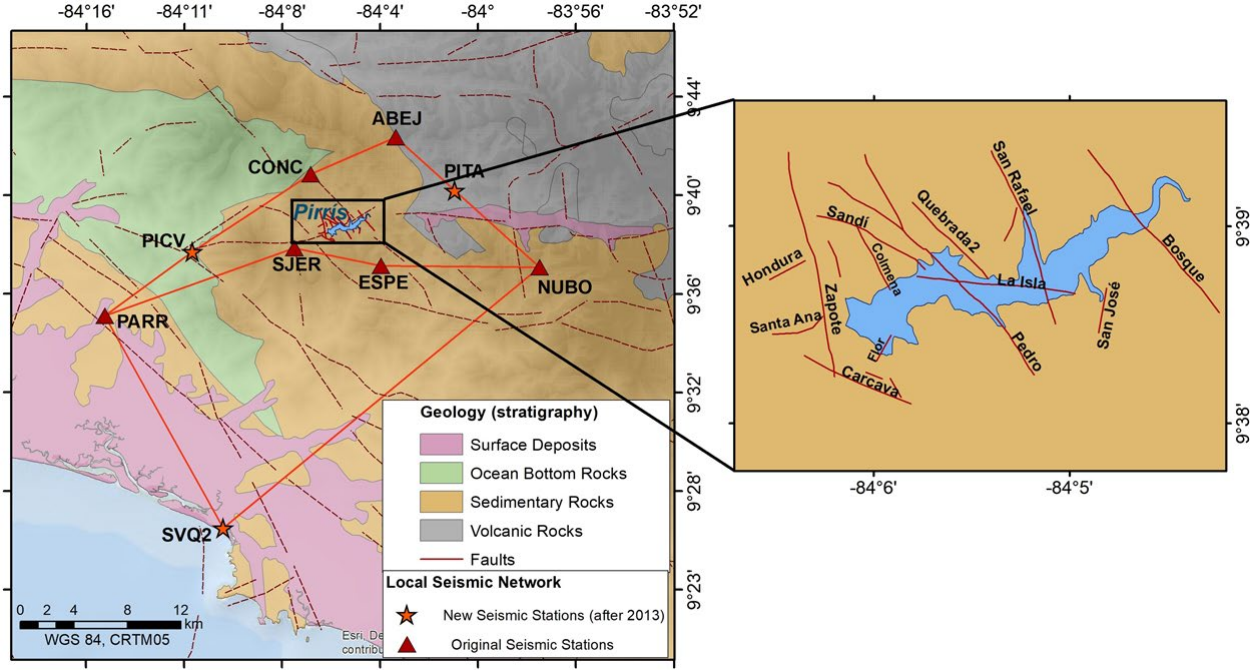
Local geological environment around the reservoir area, showing the local seismic network^39^; insert at right shows a close-up of the main local faults around the Pirrís Reservoir. This figure was generated by using the ESRI software, ArcGIS for desktop, version 10.3.1. (http://www.esri.com/).

This convergent margin along the Pacific coast, (Mid-America trench) forms a complex regional-scale shallow crustal fault system in central Costa Rica, which generates an intensive seismic activity with predominantly thrust source mechanisms. The reservoir is located in a sedimentary environment (deep marine sandstone and siltstones of Paleogene Period) surrounded by Upper Cretaceous and Paleogene igneous rocks (gabbros and pillow lava basalts) and competent Paleocene-Eocene limestones (Fig. 2). Different studies^19–21^ confirm the existence of faults within the reservoir area, which are mainly oriented in a NW-SE direction (Fig. 2), with some small fault traces oriented from NE to SW. These faults have a seismic potential of generating moderate magnitude earthquakes (4.9 < *M*_*w*_ < 6.2): the mean value of the expected maximum magnitude for each fault has been estimated by means of different empirical relationships^22–24^. What is known about the main local faults around the reservoir is summarised in Table 2 below.

**Table 2:** Characteristics of the main local active faults around the reservoir.

Fault Name	Type	Strike	Length (km)	Mmax
Zapote	Dextral	350	8	6.2
Garrobo	Dextral/Normal	340	0.8	4.9
Colmena	Dextral	340	2.4	5.5
Sandí	Dextral/Normal	310	0.6	4.7
Pedro	Dextral	350	8	6.2
Quebrada 2	Dextral	320	2.0	5.4
San Luis	Normal	30	0.8	4.9
Bosque		330	3.6	5.7
San José	Normal	10	0.9	4.9
Cárcava	Dextral	300	1.4	5.2
San Carlos	Normal	330	1.8	5.3
Paso	Reverse	110	0.4	4.5
Flor	Sinestral	15	0.6	4.7
La Isla	Normal	100	3.0	5.6
San Rafael	Sinestral	150	6.0	6.0

To date, there has been no evidence of high-magnitude historical and instrumental earthquakes (*M*_*w*_ > 6.0) associated with any local faults close to the Pirrís reservoir (at a distance <25 km). One of the most important earthquake in the area was an M 7.0 event recorded in 1952, with a maximum intensity of VII in the Pirrís dam area^25^. This event was associated with the subduction process. Accordingly, continuous seismicity monitoring (both before and after impoundment) using a dense local network is highly important in order to guarantee the safety of these critical emplacements. The availability of good quality data will help achieve a criterion for forecasting triggered seismic events in the future.

## Analysis of Seismic Data

### Seismic network and data collected

**Figure 3:**
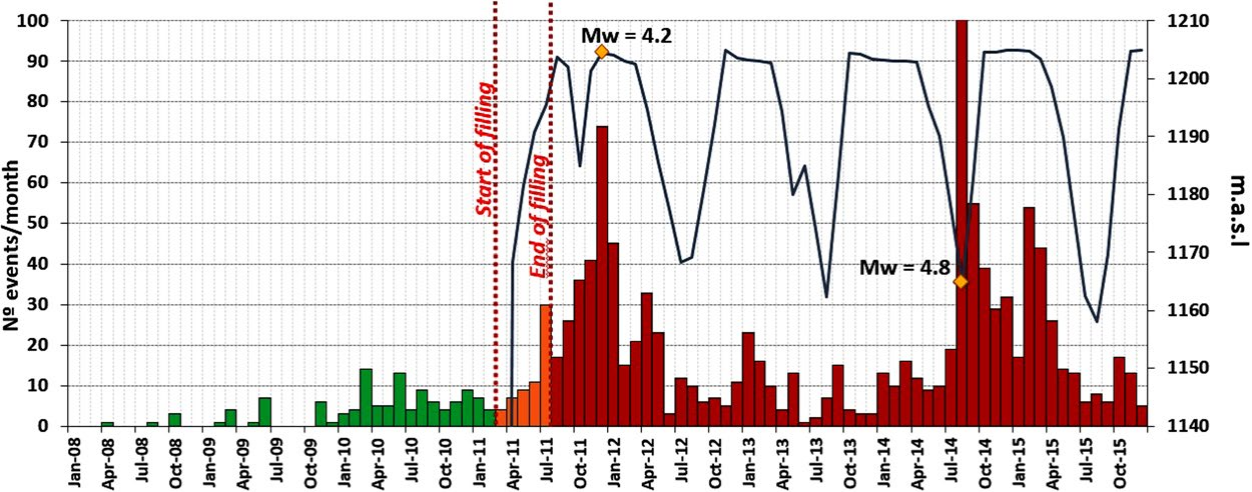
Water level variations over time in the Pirrís Reservoir (blue line) from the start of impoundment (March 2011) to December 2015. The time trend in seismicity for a 20 × 20 *km*^2^ area around the Pirrís Reservoir is shown (Seismic events with depths ≤20 km). The number of events recorded per month is shown, differentiating between ‘before’ (green), ‘during’ (orange) and ‘after’ (red).

A local seismic network was installed prior to impoundment (February 2008). Originally, the seismic network consisted of 6 broadband seismic stations. After five years, the network was densified in 2013 by the installation of three additional seismic stations, to improve the spatial coverage of the zone lying closest to the reservoir (see Fig. 2). The seismic data covered the periods before, during and after complete impoundment of the reservoir, thereby making it possible to study the three different time periods independently and compare the background seismic activity before the filling of the reservoir to the seismicity pattern recorded after its impoundment. For analysis purposes, we used all seismic events recorded from February 2008 to December 2015, considering only those with depths ≤20 km. Impoundment started on 9^*th*^ March 2011, with the Pirrís Reservoir reaching its maximum water load after 5 months, on 16^*th*^ August. Year after year, the water level of the reservoir undergoes variations of an approximately cyclical nature. The filling history shows an annual trend, with maximum water levels during the rainy season and minimum water levels when the dry season starts. World-wide studies suggest a positive correlation between large water columns in reservoirs and triggered seismicity in the area immediately surrounding them^26,27^. Figure 3 shows the temporal variation in the water level at Pirrís, together with the seismicity recorded around the reservoir. As will be seen, four annual cycles of water-level fluctuation can be differentiated. The analysis of the seismicity is explained below.

### Descriptive analysis of seismicity

In a first step, we performed a careful analysis of the seismic events which occurred in the vicinity of the Pirrís Reservoir from February 2008 to December 2015. The analysis covered the entire recorded seismicity period, with a distinction being drawn between three different periods, i.e., before, during and after impoundment, in order to identify possible changes in the seismicity pattern in the area immediately surrounding the reservoir.

The catalogue was homogenised to a common magnitude scale (*M*_*w*_) by means of the Rojas *et al*.’s^28^ two-steps relationships. The total number of analyzed events inside a 20 × 20 *km*^2^ area around the reservoir is N = 1253, ranging from *M*_*w*_ = 1.0 to *M*_*w*_ = 4.8. For study purposes, all events with depths h ≤ 20 km and locations with RMS ≤ 0.4 were considered. Most of the events have magnitudes of *M*_*w*_ [2–3] and depths of h [0–3 km].

**Figure 4:**
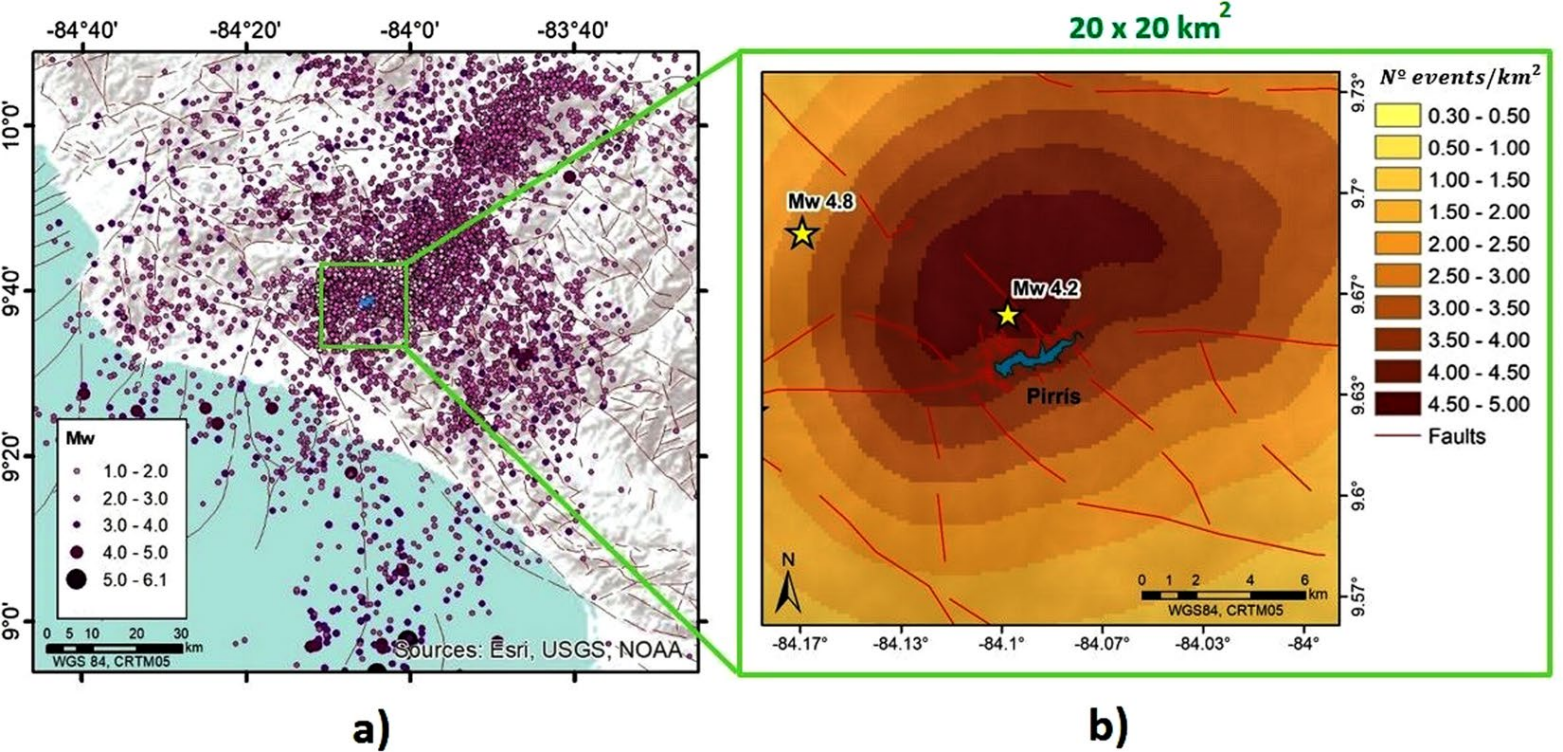
(**a**) Epicentral distribution map from February 2008 to December 2015, (**b**) Spatial distribution of seismicity represented by the *num*.*events*/*km*^2^. This figure was generated by using the ESRI software, ArcGIS for desktop, version 10.3.1. (http://www.esri.com/). Source of the base map: Image of Esri, USGS, NOAA.

By analysing the seismicity recorded within a 10 km radius of the reservoir, two events of magnitude *M*_*w*_ ≥ 4.0 were identified after the end of the initial impoundment. To date, the greatest magnitude recorded in this area was a *M*_*w*_ 4.8, corresponding to an event of h = 14 km depth and epicentral distance (d = 8.5 km) from the dam site. This event took place in August of 2014, when a complete water-unloading operation was undertaken in order to check the driving tunnel. In addition, a very shallow event (h = 2 km) with a magnitude *M*_*w*_ 4.2 was recorded in the area closest to the reservoir (at an epicentral distance of d = 2 km from the reservoir). This event occurred in December 2011, 5 months after the maximum water level had been reached. Figure 4 illustrates the epicentral distribution (Fig. 4a) and the concentration of events per area (Fig. 4b).

Due to the initial spatial dispersion of the epicentres and uncertainties surrounding the depth locations (most of the events were clustered at depths of 0–3 km), a relocation procedure was performed so as to identify possible spatial patterns or alignments of seismicity in the area closest to the reservoir.

### Relocation of events and spatial evolution of seismicity

To improve hypocentral locations and identify possible spatial patterns of seismicity, a relocation procedure was carefully carried out, using the double difference method^29^ implemented in the HypoDD programme. In this case, we considered the P- and S-seismic wave arrival times and assumed a simplified velocity structure model for the area (based on Quintero and Kissling)^30^ (Table 3). Once these events had been relocated, some spatial seismicity patterns and alignments could be appreciated near the reservoir.

**Table 3:** Simplified velocity structure model based on Quintero and Kissling^30^.

Depth (km)	*Vs* (m/s)
0	4.45
4	6.0
7	6.15
10	6.25
17	6.5
24	6.8
30	7.0

**Figure 5:**
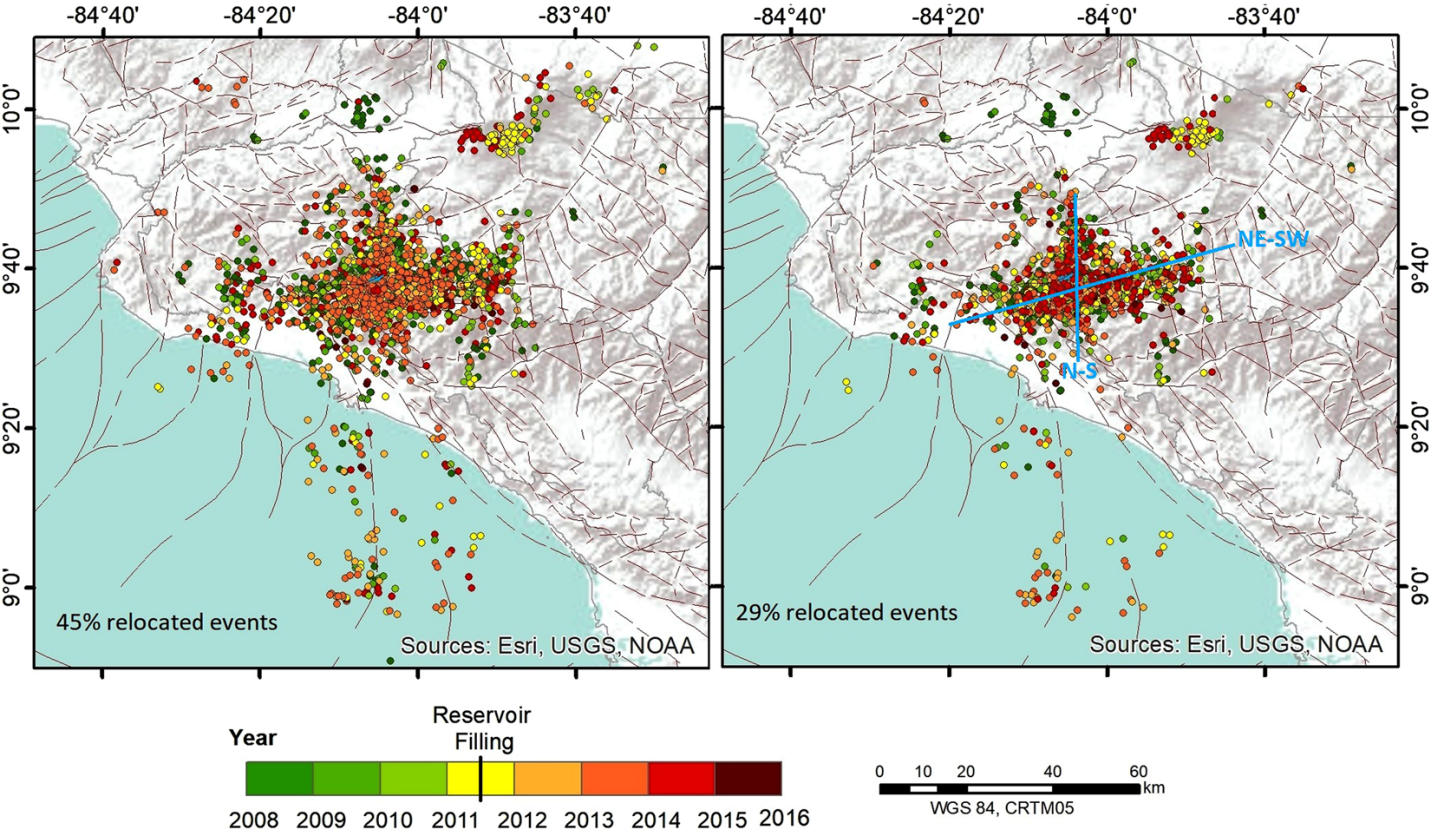
Epicentral distribution for the relocated events showing: a first relocation (left panel) in which 45% of events are relocated; and a second relocation with the 29% of events relocated. In the latter case, two seismicity alignments are more clearly noticeable. This figure was generated by using the ESRI software, ArcGIS for desktop, version 10.3.1. (http://www.esri.com/). Source of the base map: Image of Esri, USGS, NOAA.

**Figure 6:**
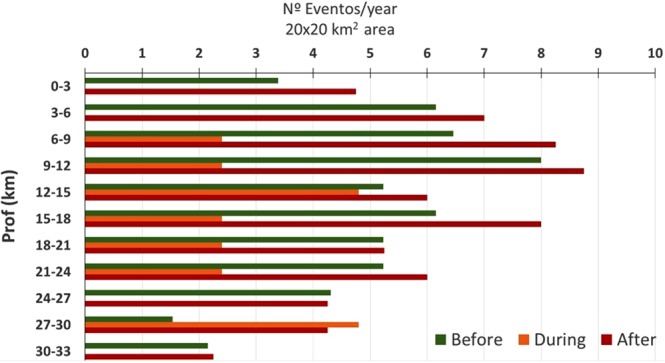
Depth distribution of relocated events.

Figure 5 shows the relocation results with a mean RMS of 0.2 (left panel) and a better solution with a lower RMS value of around 0.17 (right panel). As can be seen in both cases, two different seismicity alignments can be discerned (NE-SW and N-S). The depth distribution of the relocated events was also analysed, taking a 20 × 20 *km*^2^ area around the reservoir. The events recorded pre- and post-impoundment are differentiated by colour (red and green respectively). As can be observed from Fig. 6, the events tend to occur at depths ranging from 6 to 18 km. In general, the number of events per year increased at all depths after impoundment.

### Temporal evolution of seismicity

**Figure 7:**
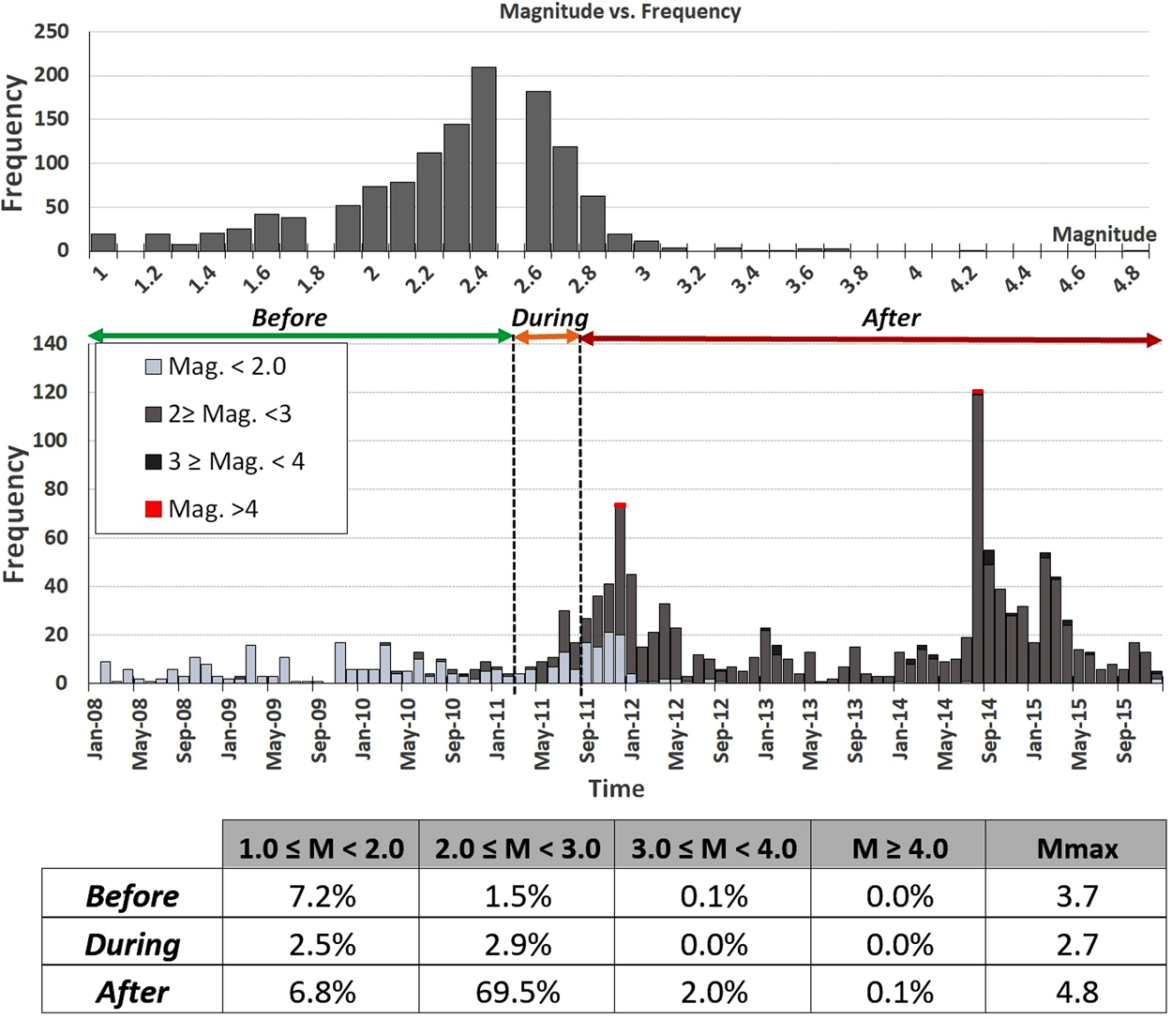
Magnitude-Frecuency plot and percentage of events with respect to the total number of events for different magnitude intervals. The occurrence time for the periods before, during and after the filling of the reservoir is differentiated by colour.

This section presents the time trend in seismicity recorded by the local network since its installation in 2008. The temporal distribution of events and its relation with monthly water-level changes (blue line) are shown in Figure 3. A considerable increase in seismicity will be seen following impoundment (red bars). The two *M*_*w*_ ≥ 4.0 events that occurred near the reservoir are depicted by yellow diamonds, in order to identify possible temporal correlations with the change in water level. Two seismic clusters appear around these two main events, probably due to the sum of the occurrence of foreshocks-aftershocks and background seismicity of this area. There are different ways of depicting the increase in the seismicity once impoundment has finished. As can be observed in Fig. 7, most of the recorded events occurred after impoundment, for a magnitude range of *M*_*w*_ 2.0–3.0. In this case, the percentage of events is shown for different magnitude ranges for each time period studied (before, during and after the reservoir filling operation) in relation to the total number of events.

Table 4 also shows the number of events for each time interval, for different magnitude ranges. In this case, instead of calculating the percentage of the total number of events, the number of events is shown for each period evaluated.

**Table 4:** Number of events for different magnitude ranges recorded before, during and after impoundment.

	Before	During	After
*M*_*w*_ ≥ 4.0	0	0	2
3.0 ≤ *M*_*w*_ ≤ 3.9	1	0	27
2.0 ≤ *M*_*w*_ ≤ 2.9	20	39	941
1.0 ≤ *M*_*w*_ ≤ 1.9	97	34	92

**Figure 8:**
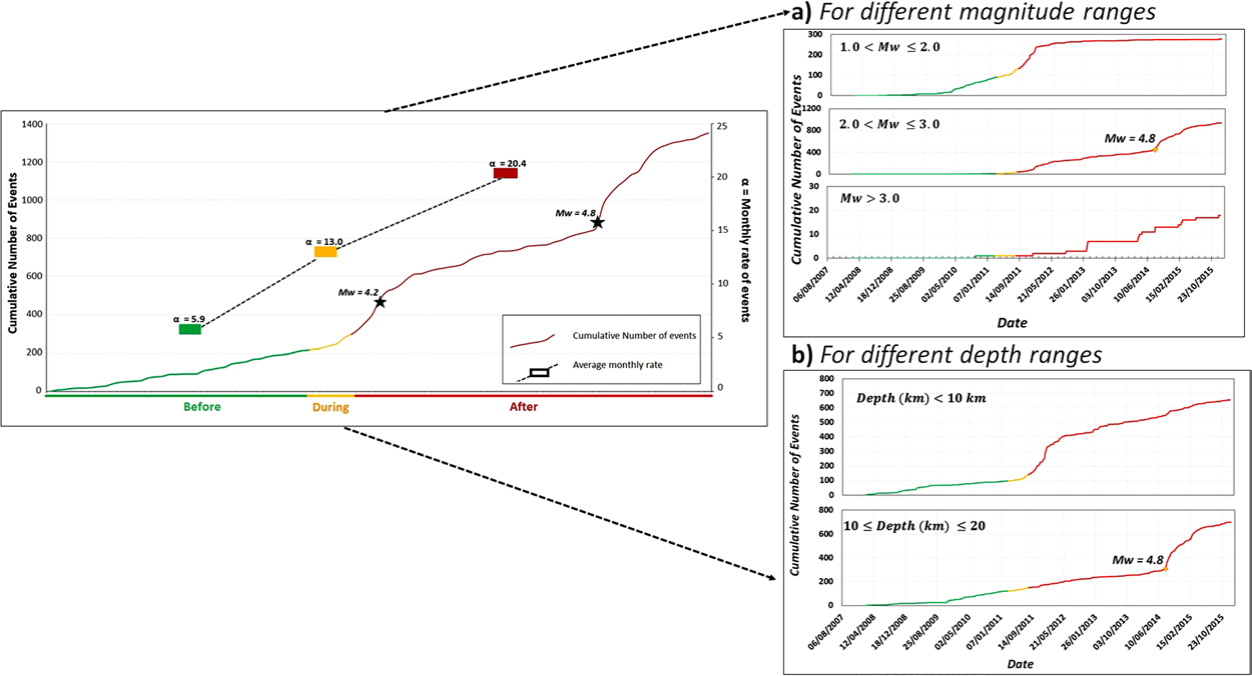
Cumulative number of events and monthly rate of events for each period evaluated (before, during and after the filling of the reservoir). Cumulative number of events over time are shown for different earthquake magnitude ranges (**a**) and depth ranges (**b**).

Additionally, we analysed the cumulative number of events and the monthly rate of occurrence of variations. Figure 8 shows a clear difference in the slope of the cumulative number of events after filling was complete (red). Similarly, a change in slope is also in evidence after impoundment. This change in the cumulative number of events corresponds to the occurrence of the *M*_*w*_ 4.8 earthquake. Moreover, there was a remarkable increase in the mean occurrence of events per month following impoundment.

**Figure 9:**
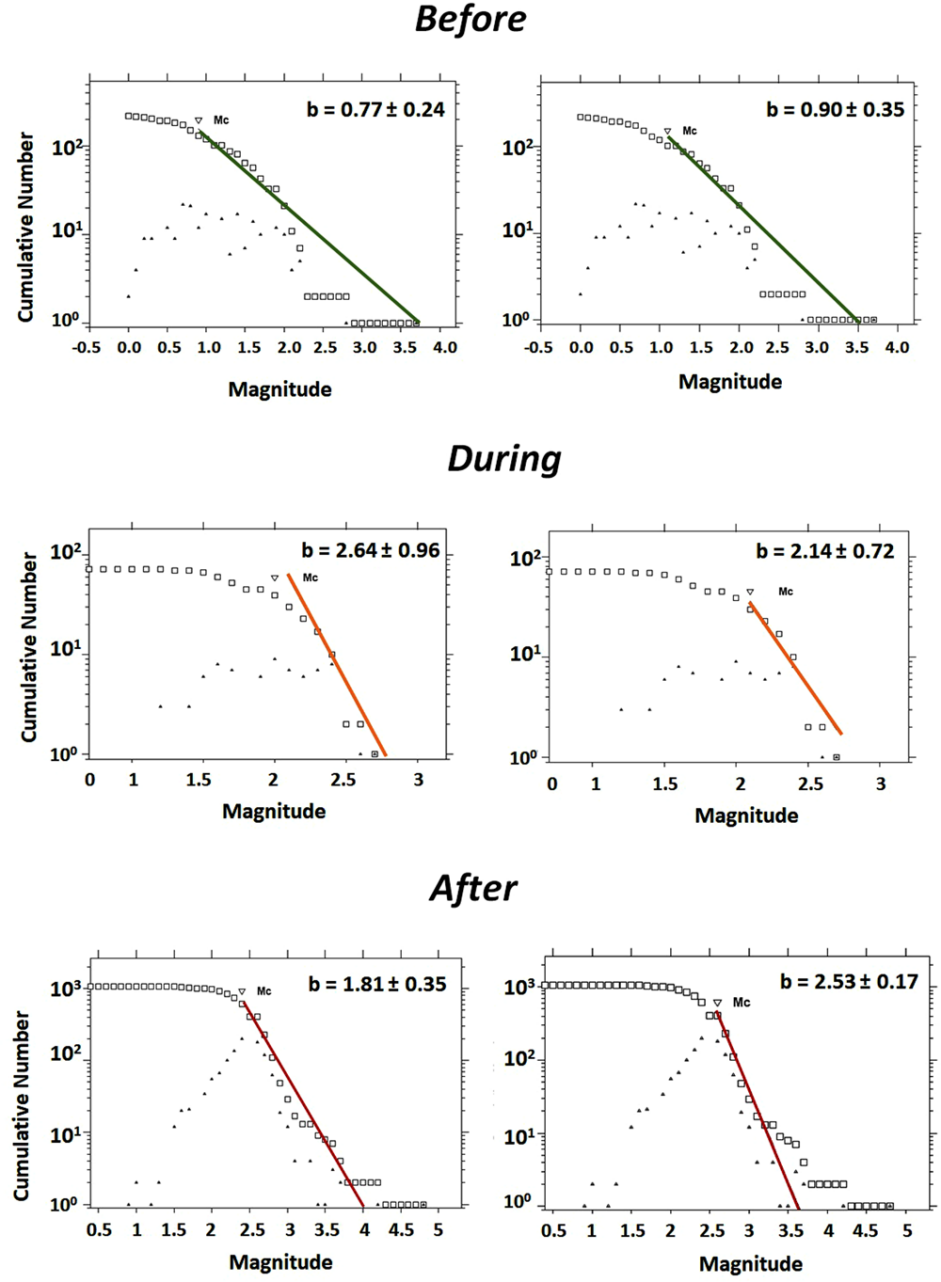
GR law for the three periods analysed, showing the solutions for the maximum curvature method (left column) and the entire magnitude range (EMR) method (right column). The figure was generated by using the Mapseis software.

Figure 9 shows the time trend for different magnitude intervals and depths. A clear increase in seismicity after the end of the filling operations can be observed, especially for magnitudes >2.0. There is a considerable increase in the occurrence of events at depths ≤10 km immediately after the end of the reservoir impoundment (see Fig. 9); however, a steep increase in seismicity below this depth is observed only after the occurrence of the *M*_*w*_ 4.8 earthquake. This increment in seismicity can be accounted for by two effects: firstly, the aftershock activity following the *M*_*w*_ 4.8 event; and secondly, a possibly delayed pore-pressure diffusion triggering effect on the seismicity at depth. Nevertheless, more detailed analyses are needed to confirm the existence of both these effects. With the aim of characterising seismicity in the area, the well-known Gutenberg-Richter (GR) law was applied. The GR relationship (log N = a-bm) represents the relationship between the number of earthquakes and their magnitude^31^, where N represents the number of earthquakes of a magnitude equal to or greater than m, a is the number of earthquakes of magnitude M = 0, and b is the slope of the line that represents the proportion between the number of earthquakes of large and small magnitudes. Table 5 summarises the GR parameters obtained for the three periods analysed, i.e., before, during and after impoundment. It is worth noting that the b-value considerably changes once the filling operations start: it evolves from a value closer to b = 1.0 (case of natural seismicity) to a higher value close to b = 2.0 (as has already been observed in other cases of induced seismicity). By applying two different methods for the adjustment, we verified that the results converge, thereby lending greater robustness to the estimates (Fig. 9).

**Table 5:** GR parameters for the three time periods studied: before, during and after impoundment.

	Maximum Curvature			EMR (Entire Magnitude Range)		
*TimePeriod*	b ± *σ*	a	*M*_*c*_ ± *σ*	b ± *σ*	a	*M*_*c*_ ± *σ*
Before	0.66 ± 0.06	3.17	0.81 ± 0.15	0.73 ± 0.11	2.81	1.0 ± 0.25
During	2.56 ± 0.96	7.44	2.2 ± 0.18	2.86 ± 0.93	8.14	2.3 ± 0.13
After	1.99 ± 0.39	8.04	2.5 ± 0.10	2.27 ± 0.10	8.82	2.6 ± 0.06

## Spatio-temporal analysis of Coulomb Failure Stress changes due to water loads

### Method and modelling

High stress changes may explain some human-induced seismicity. This can be analysed by calculating Coulomb stress changes, which in many cases can be large enough for triggering earthquakes in critically stressed faults. The Coulomb failure criterion may be expressed as^32^:

1 $${\rm{\Delta }}CFS={\rm{\Delta }}\tau +\mu ({\rm{\Delta }}{\sigma }_{n}+{\rm{\Delta }}P)$$

where Δ*τ* is the shear stress change calculated along the slip direction on the assumed fault plane, Δ*σ*_*n*_ is the normal stress change, ΔP expresses the pore pressure change, and *μ* indicates the dry friction coefficient. Hence, the Coulomb static stress changes ΔCFS expression for reservoir filling has two sources: firstly, the static loading due to the water impounded in the reservoir; and secondly, the effect of the variation in pore pressure due to the diffusion of the water loaded^33^.

Under undrained conditions in an isotropic fault zone model the Coulomb stress changes can be expressed by the following equation:

2 $${\rm{\Delta }}CFS={\rm{\Delta }}\tau +\mu ({\rm{\Delta }}{\sigma }_{n}-\frac{B}{3}{\rm{\Delta }}{\sigma }_{kk})$$

where B is the Skempton’s coefficient of the rock and *σ*_*kk*_ is the volumetric stress.

Accepting Simpson and Reasenberg’s^34^ assumption that fault zone material is more ductile than the surrounding materials^35^, then Δ*σ*_*kk*_/3 = Δ*σ*_*n*_, thus obtaining the next expression:

3 $${\rm{\Delta }}CFS={\rm{\Delta }}\tau +\mu ({\rm{\Delta }}{\sigma }_{n}\mathrm{(1}-B)$$

Where *μ*^'^ = *μ*(1 − B) is the apparent coefficient of friction, such that:

4 $${\rm{\Delta }}CFS={\rm{\Delta }}\tau -\mu ^{\prime} -{\rm{\Delta }}{\sigma }_{n}$$

The parameter *μ*′ attempts to incorporate the effects of both friction and pore pressure, and it is independent of the tectonic environment, magnitude of the stress changes and time^36^. In this study, different *μ*′ values were considered. A careful analysis was performed to understand the possible effects that water-column and water-level changes may have on the stress state of the medium. We calculated the changes in Coulomb static stress considering different input parameters. The Boussinesq solution^37^ was applied to calculate the Coulomb static stress changes due to water load. We evaluated the spatio-temporal evolution of Coulomb stress for different horizontal planes situated below the reservoir at different depths, and for different times, with a specific computer code in fortran language being developed for this purpose.

The analytical Boussinesq solution considers surface vertical static stress, assuming a homogeneous elastic half-space in a 3D tensorial form. The vertical surface forces due to water load can be calculated by means of equation 5, as follows:

5 $$F(x,y,t)=\rho gsh(x,y,t)$$

Where *ρ* = 1000 *kg*/*m*^3^, g = 9.81 *m*/*s*^2^, s is the resolution area of the Digital Elevation Model (DEM) at the site, and h (x, y, t) is the water column height in each spatial location of the reservoir for a specific time. It is important to highlight the fact that the 3D-geometry of the reservoir was taken into account when estimating the stress changes in xy-spatial planes at different depths beneath the reservoir. To do this, a DEM of the reservoir before the impoundment was generated through the level curve topography of the area before the dam’s construction. The DEM was sampled, creating a point mesh with a size/resolution of 20 × 20 *m*^2^ which covered the entire reservoir basin (bottom), thus modelling its real 3D shape. At each point of the mesh so generated, the vertical weight of the water column and its variation in time were computed.

**Figure 10:**
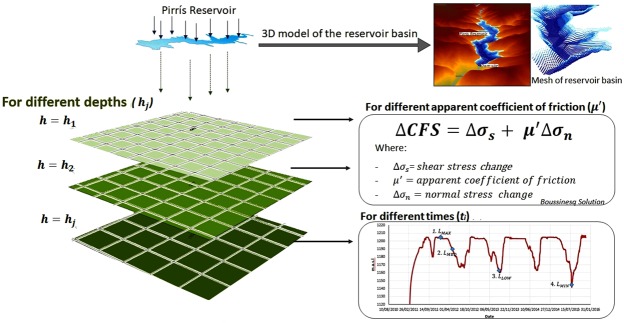
Summary of the methodology.

Furthermore, we analysed the possible influence which different input parameters might have on the final ΔCFS results. A sensitivity analysis was performed, by varying some of the input parameters while keeping the remaining variables unchanged. We assumed different saturation states for the medium, by considering apparent friction coefficients *μ*′ ranging from 0.1 to 0.9. A saturated and a dry medium were thus considered (through different intermediate cases). ΔCFS were computed for xy-planes at different depths beneath the reservoir and for different times (with each corresponding to a given water-load state of the reservoir), which meant that the spatio-temporal evolution of changes in ΔCFS was analysed by reference to surface-water load (Fig. 10).

### Spatial pattern of effective Coulomb failure stress changes

**Figure 11:**
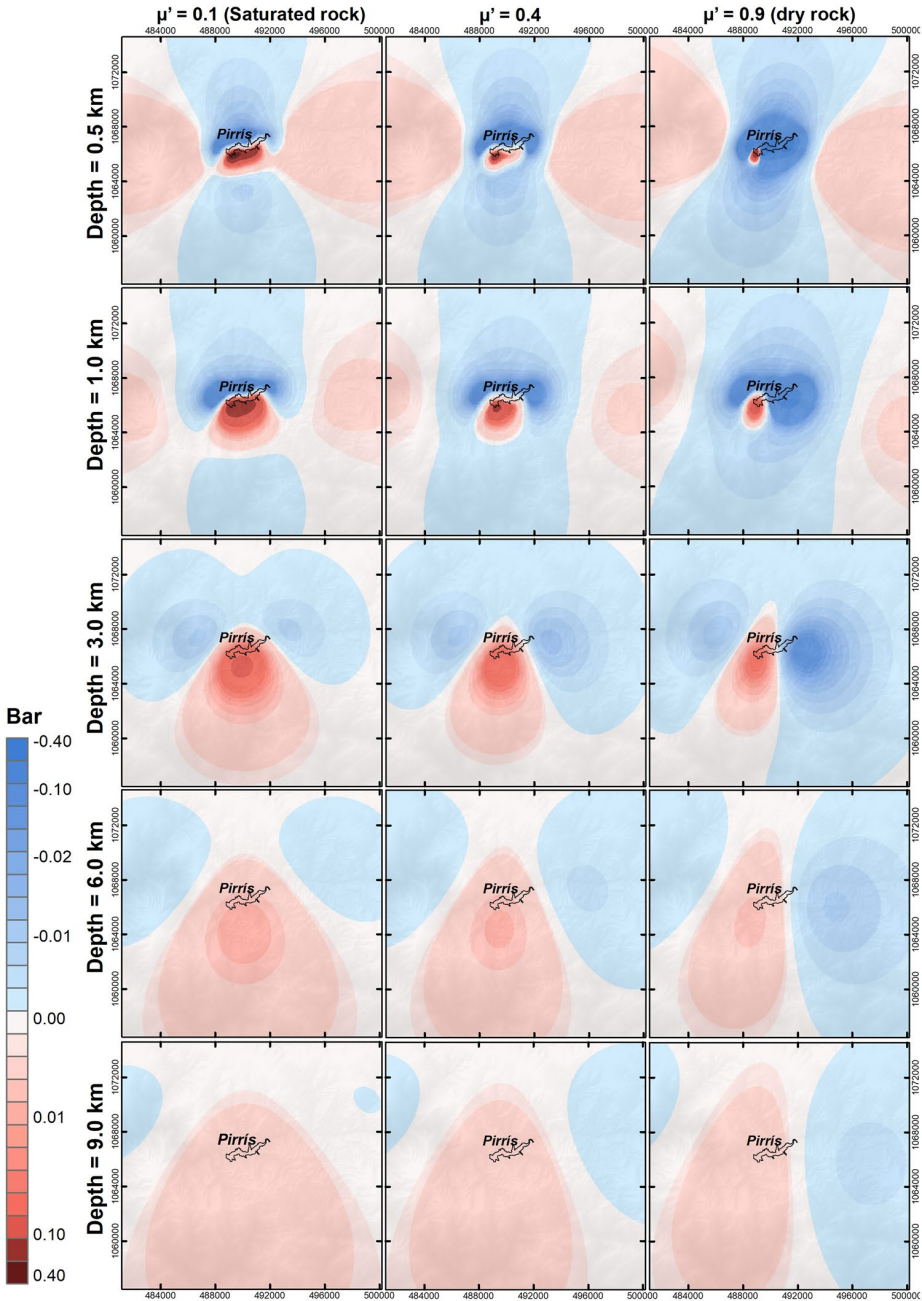
Spatial evolution of static Coulomb failure stress changes when the water load is at maximum for different depths and states of rock saturation: 20-km area around the reservoir.

At this stage, the spatial changes in Coulomb failure stress were evaluated, taking into account different apparent friction coefficients (*μ*′). We assumed a value of *μ*′ = 0.1 (rock almost completely saturated), *μ*^'^ = 0.9 (rock practically dry), and an intermediate case of *μ*′ = 0.4. The Coulomb stress changes were evaluated for a time value corresponding to the maximum water load, 1204 metres above sea level (m a.s.l.). The ΔCFS were resolved along the direction of the focal mechanism solution of the corresponding earthquake, close to the dam after its filling. The applied rotation was (str = 212, dip = 66, rak = −33). Figure 11 shows the ΔCFS results, taking into account depths beneath the reservoir ranging from 0.5 km to 10 km. All the results are expressed in bars (1Bar = 0.1 MPa). In all cases, the results show a positively loaded area in the dam site for all depths and *μ*′ considered.

As depth increases, the positive ΔCFS areas become spatially expanded towards the southern side of the Pirrís Reservoir. At shallower depths, a near field effect of positive ΔCFS concentrated can be discerned just below the reservoir, along with a more distant positive load area on the E and W sides of the reservoir. This second effect essentially disappears at depths ≥3 km. The ΔCFS values decrease with the increase in depth and *μ*′ (dry rock conditions). Independently of the depth and saturation conditions, the dam site (situated on the west side of the reservoir) is always in a positive load area. The maximum positive ΔCFS value of CFS≈0.3 bars (i.e., 0.03 MPa) is reached at the shallowest depth below the reservoir, assuming a saturated condition. The ΔCFS decreases at the same depth of 0.5 km for dry conditions, with ΔCFS 0.2 bars. As depth increases, the area of positive loading becomes spatially extended downwards, with disappearance of the lateral lobes, and smaller loads, with values lower than 0.10 bar for h > 1 km. Different authors suggest that stress changes of 0.01 MPa may be associated with previously studied RTS cases^38^. This means that the changes in the stress field around Pirrís resulting from the water-load effect may be high enough to induce earthquakes in the nearest-lying area associated with reservoir operations.

### Temporal evolution of effective Coulomb failure stress changes

Once the spatial evolution of the ΔCFS had been evaluated (assuming a maximum water level), we proceeded to analyse the temporal evolution of ΔCFS due to water-load variations. Different times corresponding to a specific reservoir water load were considered. We made an accurate estimate of the temporal evolution of ΔCFS due to changes in the water column, taking the following times into account (see Fig. 10):

1. *L*_*MAX*_: reservoir at maximum water level, i.e., 1204 m a.s.l.
2. *L*_*MED*_: reservoir at intermediate water level, 1184 m a.s.l.
3. *L*_*LOW*_: reservoir at low water level, 1165 m a.s.l.
4. *L*_*MIN*_: reservoir at minimum filling level, 1145 m a.s.l.

**Figure 12:**
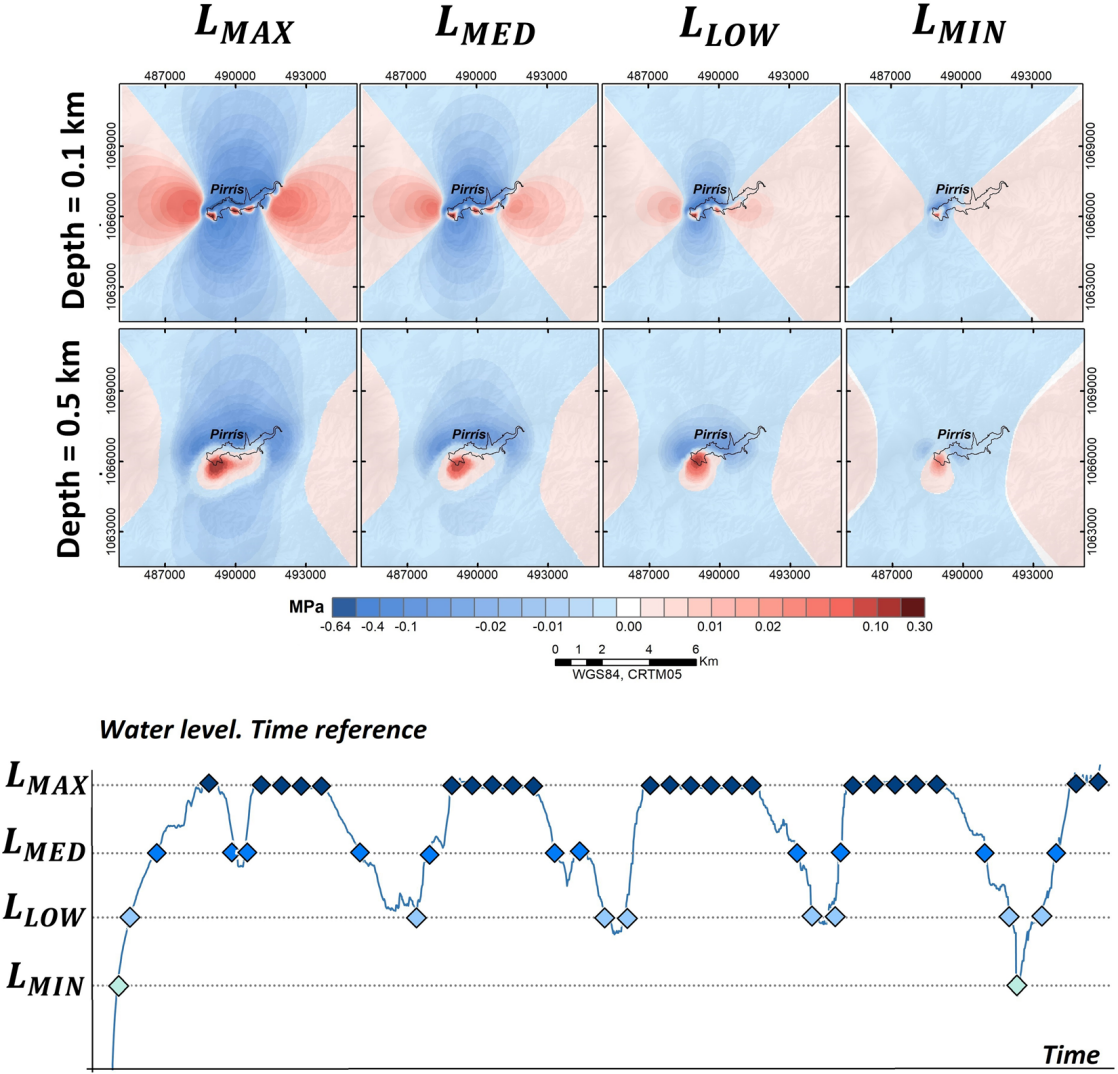
Temporal evolution of the ΔCFS for the area closest to the Pirrís Reservoir for depths ≤0.5 km: local environment (10 × 10 *km*^2^), (diamonds represent the times corresponding to each water-column level considered).
- An 20 × 20 *km*^2^ aarea with depths ranging from 0.5 to 10 km, in order to see the Coulomb stress changes distribution in a more regional environment (Fig. 13).

The following two spatial windows were selected to analyse the ΔCFS results:

- An 10 × 10 *km*^2^ area around the reservoir, in order to appreciate the nearest changes in the Coulomb stress distribution, considering very shallow depths, i.e., local environment (Fig. 12).

**Figure 13:**
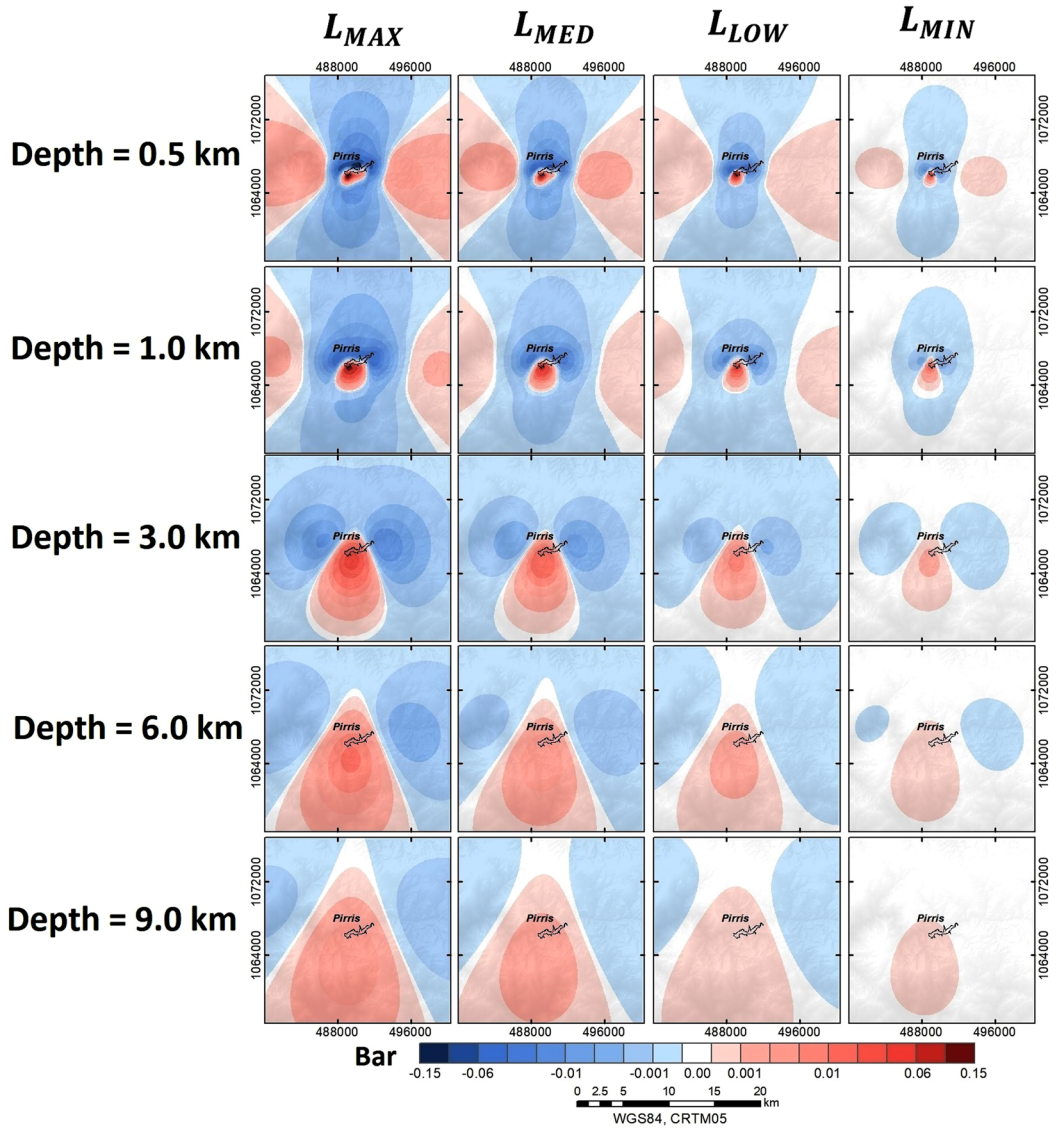
Temporal evolution of the ΔCFS in the regional environment, considering different depths <10 km: regional environment (20 × 20 *km*^2^).

In view of the results, it is important to highlight the fact that the highest ΔCFS values were obtained at the shallowest depths, when the reservoir was completely filled. In the most immediate local environment (spatial window of 10 × 10 *km*^2^), we observed that when the water load is at maximum, the near-field ΔCFS distribution at a depth of 0.1 km almost completely reproduces the shape of the reservoir, with maximum positive ΔCFS = 0.3 bar. As the water load decreases and the depth increases, however, the charged area loses its correlation with the shape of the reservoir. When a wide spatial window is considered (20 × 20 *km*^2^), it will be seen that for depths greater than 3 km, the positively charged zone extends clearly downwards and the lateral lobules disappear. In general terms, the stress pattern varies considerably in accordance with the water level, with the dam site always charged positively.

### Relationship between seismicity and Coulomb stress changes

In this environment, it is important to verify the possible correlation between water levels and the time occurrence of events. In a last step, we analysed the possible relationship between the ΔCFS and the relocated events which occurred following impoundment in the closest area. Seismicity is shown overlaid along with ΔCFS results, resolved in the direction (str = 212, dip = 66, rak = −33) and *μ*′ = 0.4. The periods when the water load is at maximum were considered in this case. For each observation plane considered in the ΔCFS estimation, the hypocentres are shown for depth intervals of ±1.0 km with respect to each observation plane. Only the events which occurred after impoundment were included.

**Figure 14:**
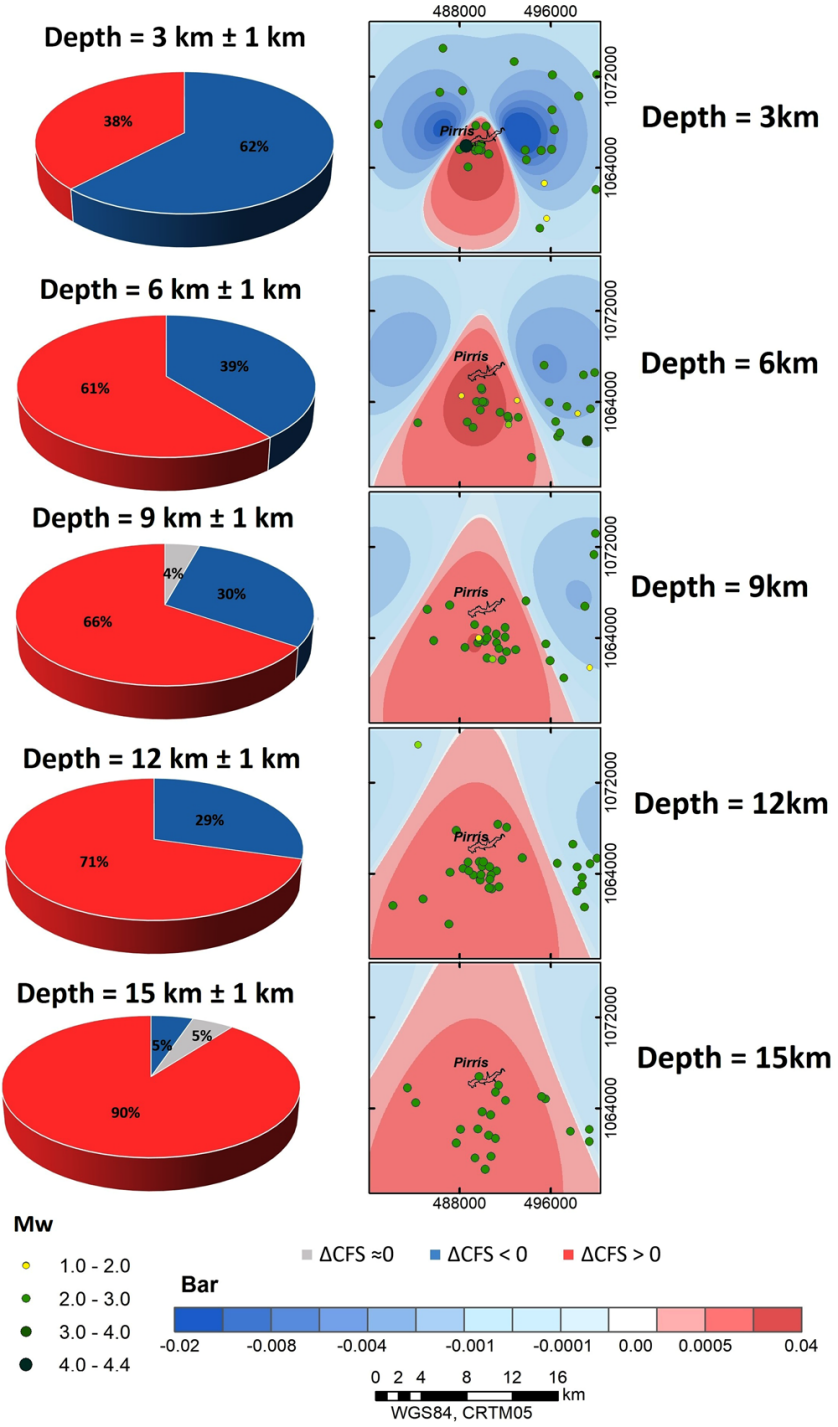
3D-view of seismicity and ΔCFS results for different depths, considering the maximum water level and a *μ*′ = 0.4.

Figure 14 shows the relationship between the hypocentral distribution and the ΔCFS for different depths below the reservoir. Here, one can appreciate a positive spatial correlation between the events and the positively charged areas for all depths, except the shallowest case (h = 3 km). Despite the fact that the greater percentage of events for this depth are in negatively charged areas, the *M*_*w*_ 4.2 event is over the positive CFS area.

Hence, some correlations between local seismicity and changes in Coulomb stress are verified. As Fig. 14 shows, the correlation between the positive areas of ΔCFS increases as depth increases. This is to be expected, as positive ΔCFS lobes increase in area, while seismicity remains in a relatively reduced horizontal area in which most of the relocated seismicity lies. This might also imply that the effects of a positive loading and a possible pre-stressed fault may be resulting in an increase in seismicity imparted by water-load changes at the surface and, above all, at the shallowest depths.

## Discussion and conclusions

While the Pirrís dam is characteristic of a medium-sized reservoir, which is probably not as large as other more significant dams with proven RTS around the world, different aspects related with the number, type and quality of the data, together with its specific geological environment, make this reservoir a special case for analysing possible induced or triggered seismicity due to reservoir filling. On the one hand, we used a unique primary dataset covering the reservoir’s operation which included: seismological data recorded using a dedicated local seismological network; the physical and geometrical characteristics of the dam; the DEM of the inundation area and the reservoir environment just before and after the beginning of operations; and data relating to filling operations, such as daily water levels, and other data pertaining to operation-related dam variations. These datasets enabled us to perform an orderly and detailed analysis of the data.

On the other hand, the reservoir is located over the mid-America subduction zone, where seismicity is linked to different types of sources, such as subduction earthquakes, intermediate depth intraplate earthquakes, and back-arc, volcanic and shallow crustal fault earthquakes. This seismotectonic environment involves the influence of this seismicity, not only on the behaviour of the dam itself, but also on the possible interaction of different seismic sources. These variables make it necessary to perform a detailed and careful analysis of the seismicity and its possible relation with the Pirrís Reservoir operation. In this study, we conducted this type of analysis plus a detailed stress-transfer analysis, including variations in space, time, depth and water saturation. The most important conclusions of these analyses are discussed below.

- Seismicity analysis.
  Following impoundment, two M_*w*_ ≥ 4.0 events were recorded: the first in December 2011, when the water level was at its maximum; and conversely, the second (M_*w*_ = 4.8) in August 2015, when the water level conditions were minimum. These events may in fact be associated with local faults, probably triggered by the reservoir filling operation. In the aftermath of these earthquakes, an increase of seismicity is to be expected due to the aftershock sequences deriving from the main events. The appreciable increase in the magnitudes of earthquakes may be indicating a positive effect of water loading over the shallow pre-stressed faults near the reservoir.
  The classification of seismicity into three different periods -before, during and after impoundment- shows clear Gutenberg- Richter differences during the periods considered. The b parameter changes from b≈1.0 before impoundment (natural background seismicity) to a higher value close to b≈2.0, clearly showing a change in the seismicity regime, usually observed in cases of induced seismicity. This change is also apparent in the magnitude time trend, where an appreciable increase is seen in events M > 2.0 and depths h > 10 km after the end of the filling operations (Fig. 9). The temporal variations in seismicity were analysed, assuming a cubic spatial volume of 20  × 20 *km*^2^ around the reservoir and a maximum depth of 20 km, in order to exclude any seismicity associated with the subduction process. Including larger depths would have produced considerable differences in the temporal analysis relating to this aspect. For its part, the event rate increased after impoundment at the shallowest depths (h ≤ 10 km), while at higher depths (h > 10 km) there were only increases in events after the *M*_*w*_ 4.8. In terms of magnitude ranges, prior to impoundment there was only background seismicity, with *M*_*w*_ (0.0–2.0); during the filling process, the event rate rose for magnitudes *M*_*w*_ (1.0–3.0); and after impoundment ended, the rate increased in both the *M*_*w*_ (2.0–3.0) and *M*_*w*_ (3.0–4.0) ranges. Indeed, the two *M*_*w*_ > 4.0 events took place during the post-impoundment period.
- Spatio-temporal analysis of Coulomb Failure Stress Changes
  An adequate analysis of seismicity and its possible relation with the reservoir operation requires a detailed computation of underground stress changes, in this case Coulomb stress change. At shallow depths, there is a clear near-field ΔCFS effect on the western side of the reservoir, due to the presence of the dam structure, and along the entire lake due to the water loads during the maximum water-level period of the reservoir; in both cases, the shape of the lake is well reproduced by a positive ΔCFS change along the preferred direction of faulting. A far-field effect is also observed for larger depths h > 10 km where the Coulomb stress changes extend laterally at greater distances. At depths equal to or greater than 3 km, the ΔCFS displays different positive and negative lobes, extending with different spatial distributions. It can be seen that the influence of positive stress farther away from the reservoir tends to disappear via lateral lobes and that the changes in positive stress become extended to the southern side of the reservoir. It is important to note that the spatial pattern of the ΔCFS is highly dependent on the stress tensor rotation applied. In this case, the focal mechanism solution assumed and used to rotate the stress tensor corresponds to one of the closest events that occurred prior to impoundment. An ideal case appears where there are detailed data on the focal mechanisms of all the potential faults around the emplacement studied.
  When the subsoil environment is dry, positive stresses concentrate around the local area of the dam. When considering different apparent friction coefficients, assuming specific values of Skempton’s coefficient, appreciable changes due to different saturation values of the medium can be observed, especially in the 10 km belt lying closest to the reservoir. As depth increases, there are fewer differences in the spatial distribution of stresses for the respective saturation conditions assumed. Values above 0.1 bar are observed for the shallowest depths. Several authors have shown that these values are usually sufficient to trigger earthquakes in the immediate environment.
  Independently of the computation depths, observation shows that for all the reservoir’s water-load conditions corresponding to different time periods, positive Coulomb stresses tend to concentrate in the area close to the dam site, especially in the western area of the reservoir. This implies that the critically pre-stressed faults loaded with positive ΔCFS stress are brought closer to failure and become more likely to produce a future earthquake in this area. A decrease in amplitude of the positive ΔCFS is also observed for times corresponding to the minimum water levels, in some cases showing a change in sign of ΔCFS, depending on such water levels. This effect is due to the combined effects of water columns along the entire surface of the reservoir. When the water load reaches its maximum level, the distribution of positive ΔCFS almost completely reproduces the shape of the lake.
- Relationship between seismicity and Coulomb Stress Changes

A positive correlation between seismicity and the ΔCFS in the area closest to the reservoir, approximately 20 km from the centroid, is clearly observable. A clear correlation between seismicity and the temporal evolution of ΔCFS is likewise observable for the first annual period of impoundment. As depth increases, the correlation between seismicity and regions of positive ΔCFS also increases. It is not evident, however, that this correlation continues for all subsequent annual periods. A second increase in seismicity is observed only 2.5 periods after the first increase. The explanation for this behaviour can be found in different effects: on the one hand, the first period of increased seismicity released most of the energy of prestressed faults near the reservoir, being most of this low seismic activity mainly represented by the inter-event times when elastic energy was being accumulated during more than one annual loading period. Once these faults accumulated enough strain along the fault interface due to tectonic loading, a second period of seismic release was then initiated during the fourth water-loading annual period. On the other hand, another possible explanation is the generation of a pore-pressure front during impoundment that may have served to inhibit seismicity production during the previous cycles. A third possible cause could be a combination of both effects wich might have inhibed seismicity during the second and third annual water-loading periods. As depth increases, the correlation between seismicity and regions of positive ΔCFS also increases.

Our analysis of the results thus reveals a clear effect of stress changes due to water-load variations. These results highlight the importance of having permanent, continuous monitoring of the seismicity associated with these types of human activities/operations, in order to guarantee the safety of these vitally important emplacements. Accordingly, studies of this nature should be undertaken in all cases of reservoir impoundment, especially where the dam in question is located in an active seismic zone with a normal fault environment. Measurements of crustal deformation and the installation of seismic stations before early impoundment operations can play a key role in understanding this type of seismicity and detecting possible changes in seismic patterns. Indeed, our case study detected: an increase in b-value after the impoundment; an increase in the rate of the shallowest events (h ≤ 10 km); a rising trend in higher-magnitude events; and a possible trigger effect on local faults. All these aspects could prove useful when it comes to controlling reservoir operations and helping take decisions aimed at guaranteeing the safety of these critical emplacements.
